# Development of a Novel Red Clay-Based Drug Delivery Carrier to Improve the Therapeutic Efficacy of Acyclovir in the Treatment of Skin Cancer

**DOI:** 10.3390/pharmaceutics15071919

**Published:** 2023-07-10

**Authors:** Arul Prakash Francis, Aftab Ahmad, Sri Durga Devi Nagarajan, Harish Sundar Yogeeswarakannan, Krishnaraj Sekar, Shah Alam Khan, Dhanalekshmi Unnikrishnan Meenakshi, Asif Husain, Mohammed A. Bazuhair, Nandakumar Selvasudha

**Affiliations:** 1 Centre of Molecular Medicine and Diagnostics (COMMAND), Saveetha Dental College and Hospitals, Saveetha Institute of Medical & Technical Sciences, Saveetha University, Chennai 600077, India; fdapharma@gmail.com (A.P.F.); shahalam@nu.edu.om (S.A.K.); 2 Health Information Technology Department, The Applied College, King Abdulaziz University, Jeddah 21589, Saudi Arabia; abdulsalam@kau.edu.sa; 3 Pharmacovigilance and Medication Safety Unit, Center of Research Excellence for Drug Research and Pharmaceutical Industries, King Abdulaziz University, Jeddah 21589, Saudi Arabia; 4 Department of Pharmaceutical Technology, Anna University, Chennai 600025, India; durga.elango10@gmail.com (S.D.D.N.); harishsundar2407@gmail.com (H.S.Y.); s.krishnapharma25@gmail.com (K.S.); 5 College of Pharmacy, National University of Science and Technology, Muscat PC 130, Oman; 6 Department of Pharmaceutical Chemistry, School of Pharmaceutical Education and Research, Jamia Hamdard, Hamdard Nagar, New Delhi 110062, India; ahusain@jamiahamdard.ac.in; 7 Department of Clinical Pharmacology, Faculty of Medicine, King Abdulaziz University, Jeddah 21589, Saudi Arabia; obazohair@kau.edu.sa; 8 Department of Biotechnology, Pondicherry University, Puducherry 605014, India

**Keywords:** acyclovir, red clay, drug release, cytotoxicity, in silico

## Abstract

Acyclovir (ACV) is a promising candidate for drug repurposing because of its potential to provide an effective treatment for viral infections and non-viral diseases, such as cancer, for which limited treatment options exist. However, its poor physicochemical properties limit its application. This study aimed to formulate and evaluate an ACV-loaded red clay nanodrug delivery system exhibiting an effective cytotoxicity. The study focused on the preparation of a complex between ACV and red clay (RC) using sucrose stearate (SS) (nanocomplex F1) as an immediate-release drug-delivery system for melanoma treatment. The synthesized nanocomplex, which had nanosized dimensions, a negative zeta potential and the drug release of approximately 85% after 3 h, was found to be promising. Characterization techniques, including FT-IR, XRD and DSC-TGA, confirmed the effective encapsulation of ACV within the nanocomplex and its stability due to intercalation. Cytotoxicity experiments conducted on melanoma cancer cell lines SK-MEL-3 revealed that the ACV release from the nanocomplex formulation F1 effectively inhibited the growth of melanoma cancer cells, with an IC_50_ of 25 ± 0.09 µg/mL. Additionally, ACV demonstrated a significant cytotoxicity at approximately 20 µg/mL in the melanoma cancer cell line, indicating its potential repurposing for skin cancer treatment. Based on these findings, it can be suggested that the RC-SS complex could be an effective drug delivery carrier for localized cancer therapy. Furthermore, the results of an in silico study suggested the addition of chitosan to the formulation for a more effective drug delivery. Energy and interaction analyses using various modules in a material studio demonstrated the high stability of the composite comprising red clay, sucrose stearate, chitosan and ACV. Thus, it could be concluded that the utilization of the red clay-based drug delivery system is a promising strategy to improve the effectiveness of targeted cancer therapy.

## 1. Introduction

Cancer is a group of complex diseases and one of the major causes of death worldwide. Among the several types of cancers, skin cancer specifically refers to the abnormal growth of skin cells. It commonly develops in areas where the skin is exposed to UV radiation, such as the scalp, face, ears, neck, arms and hands. According to the GLOBOCAN report, around 0.32 million individuals were diagnosed with skin cancer worldwide in 2020, with the highest overall rate of skin melanoma being reported in the New Zealand and Australian populations [1]. In India, the north and northeastern regions have been reported to show the highest incidences for both males and females [2]. Various treatment strategies are employed to treat melanoma, including surgery, photodynamic therapy, immunotherapy, virotherapy, targeted therapy, drug therapy, radiation therapy and chemotherapy. The drugs are generally applied as topical ointments for skin cancers. However, the current drugs used for the treatment of skin cancer lack efficacy as a result of toxicity and drug resistance. Therefore, it is crucial to find a suitable candidate that is more site-specific and less toxic to improve treatment outcomes.

The US FDA is increasingly focusing on drug repurposing due to the high risk, cost and slow pace of the development of new drugs. Recently, in the COVID-19 pandemic, many existing drugs were repurposed and tested for their efficacy in SARS-CoV-2 infection [3]. Researchers are now exploring the use of antiviral drugs to treat various cancers, as they have shown potential in suppressing cancer cell proliferation [4]. Acyclovir (ACV), an anti-viral drug commonly used to treat oral sores caused by the herpes virus [5], is being repurposed to treat skin cancer. Shaimerdenova et al. reported that ACV decreases the proliferation of cancer cells and upregulates the apoptotic pathway via cytokine caspase-3 [6]. Although ACV has various biological effects and is marketed in various dosage forms, it has a very low bioavailability (15–30%). It is classified as a BCS class III drug and has a poor permeability, which is attributed to its low bioavailability and slightly water-soluble nature [7,8]. Its existence in six different forms (four anhydrous and two hydrous) further limits its aqueous solubility. Hence, it is of paramount importance to develop a drug delivery system that can enhance the poor physicochemical properties of ACV and similar active pharmaceutical ingredients (APIs) to improve its therapeutic efficiency.

Among the various drug delivery systems, clay-based drug delivery systems have gained the attention of researchers due to the excellent properties exhibited by clay minerals, such as a low density, high porosity, large surface area, biocompatibility, etc. In particular, nanoclays and their composites with other polymers have been shown to have versatile applications in novel drug delivery systems. These composites offer a range of advantages, such as a high payload, extended stability, stimulus-responsive drug release and enhanced biodegradation. Due to the various interaction patterns between clay and API, like hydrogen bonding, cationic exchange, electrostatic interaction and hydrophobic affinity, nanoclay-based drug delivery systems offer high drug loading. Among the different types of clay, the cationic clay montmorillonite has a higher adsorption capacity than illite and kaolinite due to its specific surface area, basal interlayer spacing and greater cation exchange capacity [9]. Red clay (RC), a natural material rich in minerals, like iron, aluminum and silica [10], has not been extensively explored in drug delivery systems. However, it has been used for centuries for medicinal purposes due to its antibacterial, antifungal and anti-inflammatory properties [11]. Other clays have been well studied in treating or managing many diseases, including diabetes, colitis, inflammation, cancers, etc. Their application in melanoma has been extensively researched, and hence this study aimed to explore the anticancer activity of a red clay–sucrose stearate-based complex.

Moreover, among many natural hydrophilic polymers, chitosan, a polycation, forms bonds with anionic clay through electrostatic interactions [12], enabling high payloads of APIs, like ACV. The amino groups in chitosan can be exploited for a controlled drug release and mucoadhesion [13], allowing it to deliver drugs at the skin’s pH [14]. The complexation between RC, SS and ACV with chitosan was analyzed using in silico methods, and the results are reported here. Therefore, this study focused on formulating a novel combination of ACV, SS and RC that is more effective than ACV alone against melanoma.

## 2. Materials and Methods

### 2.1. Materials

RC was purchased online (Amazon, India). ACV with 98% purity, SS with 97% purity and all other solvents of an analytical grade were purchased from Sigma Aldrich, Bengaluru, India.

### 2.2. Methodology

#### 2.2.1. Preparation of RC-SS Complex

About two grams of RC was mixed with 50 mL of distilled water and stirred for 12 h at room temperature (RT) to obtain the suspension. Approximately 5 mL of a 5% SS solution was preheated (60 °C) and slowly added to the suspension (preheating of SS enhanced the mixing). The suspension was stirred for 5 h under magnetic stirring (1000 RPM at RT) to ensure uniform mixing and complex formation. The suspension was washed with excess distilled water to remove the unbound SS.

#### 2.2.2. Preparation of Nanocomplex F1 (ACV-Loaded RC-SS Complex)

About 10 mL of acidic ACV (20 mg/10mL of diluted HCl) was added slowly to the above suspension and stirred at room temperature for 6 h under magnetic stirring (800 RPM) to facilitate uniform drug binding. The suspension was then centrifuged at 10,000 RPM for 15 min. The pellets were dried at room temperature and used for further studies. Free ACV in the supernatant was calculated using a UV-Vis spectrophotometer.

#### 2.2.3. Characterization of Nanocomplex F

The particle size and zeta potential of the samples were measured by dispersing 0.1 mg of the samples in 1 mL of deionized water using Zetasizer (Malvern). The % encapsulation efficiency, % drug loading and % yield of the nanocomplex were determined using UV-Visible spectrophotometry.

(1) $$\%\;Encapsulation\;efficiency=\frac{Amount\;of\;ACV\;added\;initially-Free\;ACV\;in\;the\;supernatant}{Amount\;of\;ACV\;added\;initially}\times 100$$

(2) $$\%\;Drug\;loading=\frac{Amount\;of\;ACV\;entrapped\;within\;the\;nanocomplex}{Weight\;of\;nanocomplex}\times 100$$

(3) $$\%\;Yield=\frac{Weight\;of\;nanocomplex}{The\;whole\;weight\;of\;RC,SS\;and\;ACV\;added}\times 100$$

The functional groups in the compounds and their chemical interaction were obtained from Fourier-transform infrared (FTIR) spectra recorded in the range 400–4000 cm^−1^ using an FTIR spectrophotometer (Agilent Resolutions pro). The mineral composition of the samples was identified using the powder X-ray diffraction technique (XRD) at room temperature with the 2θ value of 5–80°. XRD gave insights into the interlayer variation in the sample. The thermal stability of the sample was investigated using DSC-TGA (Differential Scanning Calorimetry–Thermogravimetric analysis). A heating range of 50–800 °C with an increase in the temperature of 10 °C/min was employed under a nitrogen atmosphere (35 mL/min).

#### 2.2.4. In Vitro Permeation Study of ACV-RC-SS Complex

The in vitro release study was conducted in a vertical Franz diffusion cell with an acceptor compartment (25 mL) and a donor compartment (10 mL) separated from each other by a regenerated cellulose membrane with a molecular weight cut-off at 10-12 kDa. About 10 mg of the sample (ACV and Nanocomplex F) was added to 10 mL of a pH 5.5 phosphate buffer (skin pH) and poured into the donor compartment. The experiments were performed in triplicate, at 37 °C in the pH 5.5 phosphate buffer. Samples were periodically withdrawn from the acceptor compartment using a 1 mL pipette and the volume of the withdrawn samples was replaced with an equal volume of the fresh buffer. Then, ACV released from nanocomplex F collected from the acceptor compartment was estimated using a UV-Vis spectrophotometer at 254 nm. The amount of ACV released after permeation through the membrane was calculated from the standard calibration curve of ACV obtained before the studies.

#### 2.2.5. Interaction Study with In Silico Docking

The interaction studies of drug and red clay components were performed using two major simulation platforms. BIOVIA material studio (v 17.1.0.48) was used for geometric optimization, theoretical cell formation and mixing of various composition analyses. BIOVIA Discovery Studio (v 17.1.0.1643) was utilized to evaluate the interaction between the drug and red clay components.

#### 2.2.6. Forcite Protocol

These classical molecular mechanic tools mainly perform energy calculations and geometry optimizations for single molecules and periodic systems. In this study, 12 components of RC (Table 1) and three drugs were submitted to forcite calculation. The force field Condensed-phase Optimized Molecular Potentials for Atomistic Simulation Studies (COMPASS) were fixed and the summation of electrostatic and van der Waals by other atom-based parameters was adjusted, as given in Figures S1 and S2 (Supplementary Data).

##### Amorphous Cell Protocol

The amorphous cell built three-dimensional (3D) periodic structures of red clay and the drug system using a Monte Carlo fashion and minimizing close contact between the atoms. Twelve components of RC were loaded in the composition table (Table 1) based on the percentage. Figure S3 shows the other parameters for the cell construction (Supplementary Data).

##### Blend Protocol

The blend module aided in forecasting the miscibility of a polymer with other chemical components. The traditional experimental tactic is screening more different formulations to catch an ultimate formulation that meets all desires. Materials Studio tools of this module provide a desktop AI solution that delivers the necessity of wet lab experimentation and, more prominently, the risk of product failure. In this work, the blend task is fixed as mixing and the quality of work is set as fine. All the molecules were loaded into the input table and the role of drugs, and 12 components of red clay were fixed as base + screen. Figure S4 illustrates the blend mixing options and the values fixed for the run (Supplementary Data). After the run, the blend algorithm created a six-result file in the name of blend mixing. Blend binding energy distribution and chi values for base, polymer and drug mixtures were computed and plotted to evaluate the stability of blends.

#### 2.2.7. Cell Culture Maintenance

Human skin cancer SK-MEL-3 cell lines were procured from the National Centre for Cell Sciences (NCCS), Pune, India. Dulbecco’s Modified Eagle Media (DMEM) used for maintaining the cell line was supplemented with 10% Fetal Bovine Serum (FBS). Penicillin (100 U/mL) and streptomycin (100 μg/mL) were added to the medium to prevent bacterial contamination. The cell lines were maintained in a humidified environment with 5% CO_2_ at 37 °C.

#### 2.2.8. Cytotoxicity Studies

The cytotoxicity of doxorubicin, RC, ACV and RC+ACV in SK-MEL-3 cells was determined using the method reported by Mosmann (1983) [15]. Briefly, SK-MEL-3 cells were cultured using a DMEM medium, harvested and used for the cytotoxic assay. About 1 × 10^4^ cells/mL was seeded in each well of 96-well plates and incubated for 24 h to allow attachment. After 24 h, cells were treated with different concentrations of RC (10 to 60 μg/mL), ACV (5 to 30 μg/mL) and RC+ACV (5 to 30 μg/mL) and doxorubicin (0.5 to 25 μg/mL), and incubated at 37 °C in a humidified 95% air and 5% CO_2_ incubator for 24 h. Untreated cells were used as a control. After 24 h incubation, MTT (5 mg/mL in PBS) dye was added to each well and incubated for another 4 h at 37 °C. The purple formazan crystals formed were dissolved in 100 µL of DMSO and the absorbance was measured at 570 nm using a multi-well plate reader. The results were expressed in percentage cytotoxicity of treated cells concerning the control.

$$Cytotoxicity\;\left(\%\right)=\frac{Absorbance\;of\;Control-Absorbance\;of\;Test}{Absorbance\;of\;Control}\times 10$$

The IC_50_ values were calculated from the dose-responsive curves of doxorubicin, RC, ACV and RC+ACV, where the inhibition of 50% cytotoxicity compared to vehicle control cells. All experiments were performed in triplicate.

#### 2.2.9. Measurement of Apoptotic Induction Using Acridine Orange/Ethidium Bromide (AO/EB) Dual Staining Method

To detect apoptotic cells with condensed chromatin, the acridine orange (AO) and ethidium bromide (EB) dual staining method was employed as per Arjunan et al. (2021) [16]. Briefly, 1 × 10^5^ cells/mL of SK-MEL-3 cells was seeded in 24-well plates and was with sub-optimal concentrations of doxorubicin, RC, ACV and RC+ACV for 24 h. After the treatment, apoptotic cells took up EB, emitting red/orange fluorescence under 550 nm. Acridine orange, a DNA-selective and membrane-permeable fluorescent cationic dye entered normal cell nuclei and emitted green fluorescence under 525 nm.

### 2.3. Statistical Analysis

The values are expressed as mean ± SD. The statistical comparisons were performed with a one-way analysis of variance (ANOVA) followed by a Duncan’s Multiple Range Test (DMRT), using SPSS version 12.0 for Windows (SPSS Inc. Chicago, IL, USA; http://www.spss.com (accessed on 30 May 2023). The values were considered statistically significant if the *p*-value was less than 0.05.

## 3. Results

Clay molecules have been extensively used for various purposes in pharmaceutical industries due to their versatile functions. For the first time, red clay was analyzed for its drug carrier property and the results are reported here.

### 3.1. Characterization of the Formulation

The particle size and zeta potential analysis of RC particles showed a mean hydrodynamic diameter of 938.0 ± 97.5 nm and zeta potential of −19.0 ± 7.6 mV, respectively (Table 2). The negative charge may be attributed to the presence of higher hydroxides present in the red clay. The diameter was reduced to 426.0 ± 31 nm in nanocomplex F on the addition of SS and ACV to the suspension. Moreover, the nanocomplex suspension showed a zeta potential of −22.2 ± 5.1 mV followed by the addition of negatively charged SS and ACV. The % encapsulation efficiency, % drug loading and % yield of nanocomplex F1 were found to be 98.3 ± 6.6%, 31.10 ± 2.7% and 59.0 ± 8.2%, respectively.

**Figure 1:**
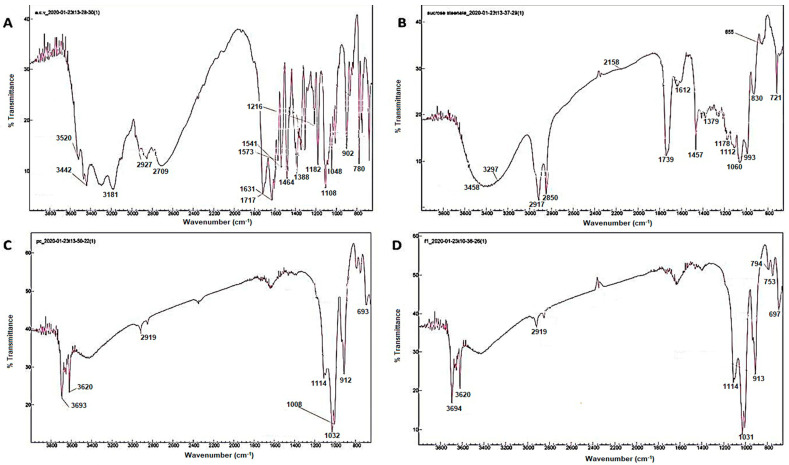
FTIR spectra of ACV (**A**), SS (**B**), RC (**C**) and nanocomplex F1 (**D**).

The FTIR results of the RC samples shown in Figure 1C revealed multiple peaks confirming clay minerals. The stretching at 912 cm^−1^ indicates the presence of Al-OH and Fe-OH; in addition, the existence of Al-Si-O was confirmed by the peak at 460 cm^−1^. The stretching at 1092 cm^−1^ and 692 cm^−1^ corresponds to the Si-O bond. The band at 3395 cm^−1^ showing a broad and low intensity may be due to the presence of water traces. In the FT-IR spectrum of ACV (Figure 1A), peaks at 3520 cm^−1^ were attributed to –OH stretching vibration. Peaks at 1631 and 3181 cm^−1^ were due to C=O stretching and -NH_2_, respectively. The C=N and C–N stretching vibrations were confirmed by the peaks at 1484 and 1182 cm^−1^, respectively. In the case of sucrose stearate, peaks at 3468 cm^−1^ and 1739 cm^−1^ were assigned to –OH stretching due to valence vibrations of the ester (νC=O), respectively (Figure 1B). The FT-IR of nanocomplex F1 (Figure 1D) was similar to the FT-IR spectrum of red clay and did not show any peaks of ACV. This confirms the effective encapsulation of ACV within the clay complex.

**Figure 2:**
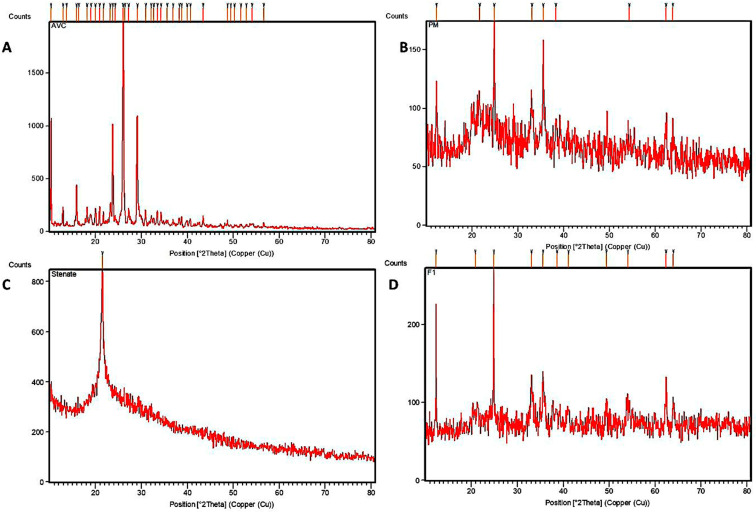
XRD pattern of ACV (**A**), RC (**B**), SS (**C**) and nanocomplex F1 (**D**).

In Figure 2A, the XRD of ACV shows a 2θ value at 8.5, 11.6, 14.56, 19.7, 22.0, 23.4, 28.74, 34.2, 40.0 and 49.05°. The RC showed peaks at 6, 27.5, 30.2, 35.5 and 37° (Figure 2B). SS revealed a peak at 20° corresponding to the peak of stearate (Figure 2C). The nanoformulation showed peaks similar to that of red clay, confirming the effective encapsulation of ACV within the RC-SS carrier. The decreased peak intensity indicated the addition of ACV on the interlayers of the RC-SS complex (Figure 2). Moreover, it provides evidence for the incorporation of drug molecules into RC through an ion exchange mechanism. 

**Figure 3:**
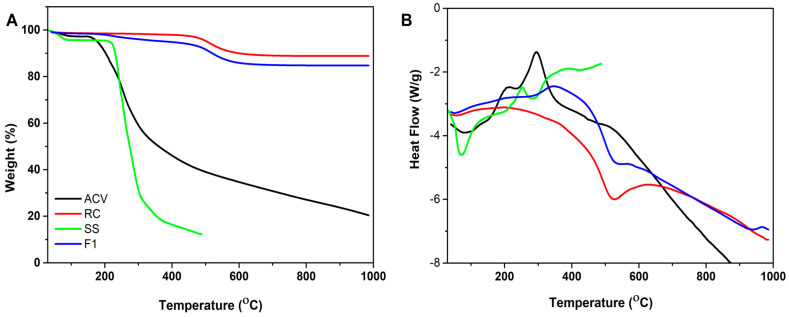
TGA overlay showing thermal degradation (weight loss (**A**) and heat flow (**B**)) of ACV, RC, SS and nanocomplex F1.

Figure 3 shows the thermal behavior of ACV, RC, SS and nanocomplex F1. The thermogram of ACV revealed an endothermic peak at 253.30 °C followed by an exothermic peak proximately, which confirmed the initiation of thermal degradation at 295.34 °C with a weight loss of 31.10%. The thermogram of RC indicated an endothermic peak at 520.36 °C corresponding to dehydration with a weight loss of about 8.47% and no degradation peak was evidenced. The sucrose stearate thermogram was characterized by an endothermic peak at 222.35 °C and degradation occurred at high temperatures with a weight loss of about 67.85%. The thermogram of nanocomplex F1 showed an exothermic peak at 368.16 °C, confirming the increase in the degradation temperature of the ACV-loaded RC-SS complex from 295.34 °C and its thermal stability. The presence of RC enhanced the thermal stability of nanocomplex F1 with a weight loss of only about 7.66%.

### 3.2. In Vitro Permeation Study of ACV-RC-SS Complex

**Figure 4:**
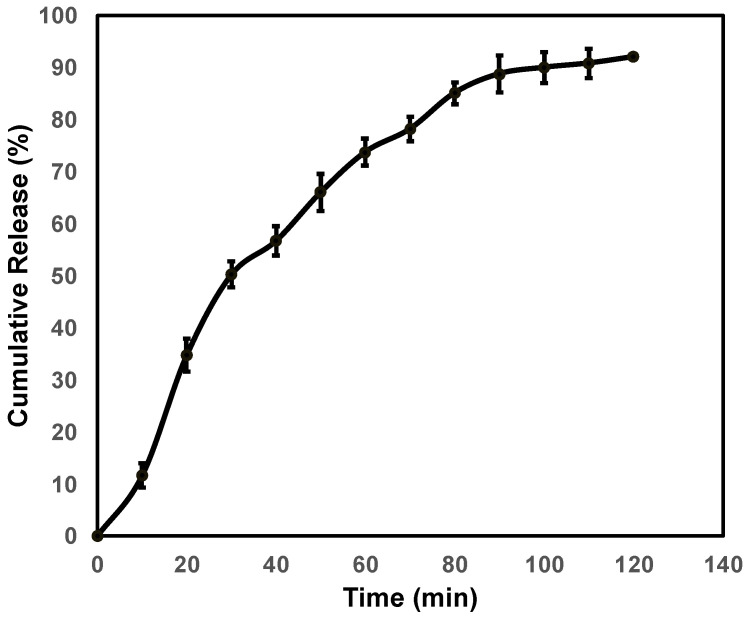
In vitro release profile of ACV from nanocomplex F1.

The in vitro study was carried out at pH 5.5 to determine the rate of the drug release after permeation through the membrane using a Franz diffusion cell. The results are given in Figure 4. The nanoclay complex showed an immediate release of ACV up to 1 h followed by a sustained release. The release slowed down as the time increased and around 90% of the drug was released after permeation at the end of 120 min. This represents an enhanced permeation and release of ACV, a BCS class III drug. The drug release after permeation at acidic conditions provides a further conclusion that the release of the drug is due to ion exchange and due to alteration in the pH.

### 3.3. Drug Release Kinetics

**Figure 5:**
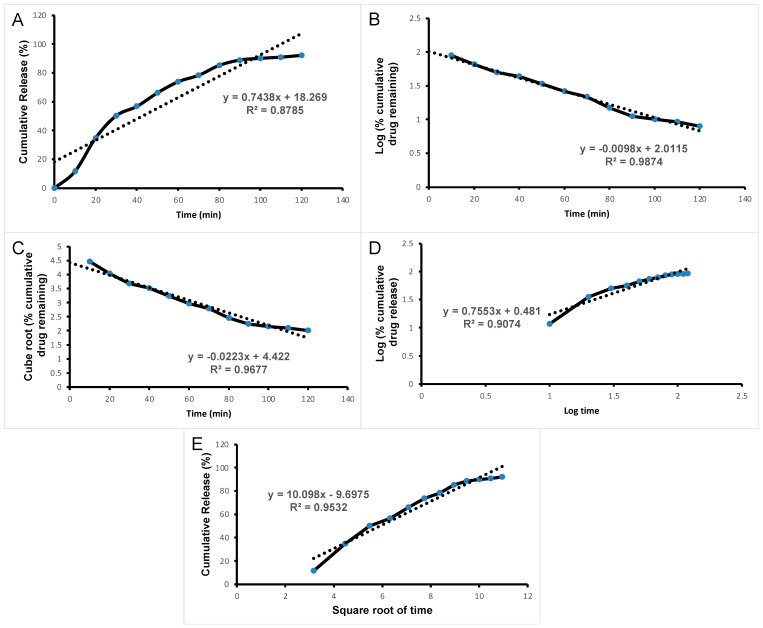
Release kinetics of ACV from the nanocomplex (**A**) Zero-order, (**B**) First-order, (**C**) Hixson–Crowell, (**D**) Krosmeyer–Peppas, (**E**) Higuchi model.

The release kinetics of ACV release (i.e., the order and the mechanism of release) from nanocomplex F1 could be inferred with different kinetic models such as the Zero-order, First-order, Hixson–Crowell, Korsmeyer–Peppas and Higuchi’s model, as given in Figure 5. From the results, it is clear that the First-order plot exhibited a good linearity (R^2^ = 0.9874) in comparison to the Zero-order plot (R^2^ = 0.8785) and ACV release from nanocomplex F also showed the best fit with the Hixson–Crowell plot with a good linearity value (R^2^ = 0.9834), supporting the dissolution of the carrier. The slope value of the Korsmeyer–Peppas plot is 0.7553, which is between 0.45 and 0.89, indicating that the drug release followed a non-fickian transport mechanism. Moreover, the Higuchi plot with a good linearity (R^2^ = 0.9532) suggested that the drug release is owing to a diffusion mechanism. Hence, combined mechanisms of diffusion, dissolution and erosion were suggested for the ACV release.

### 3.4. Interaction Study with In Silico Docking

**Figure 6:**
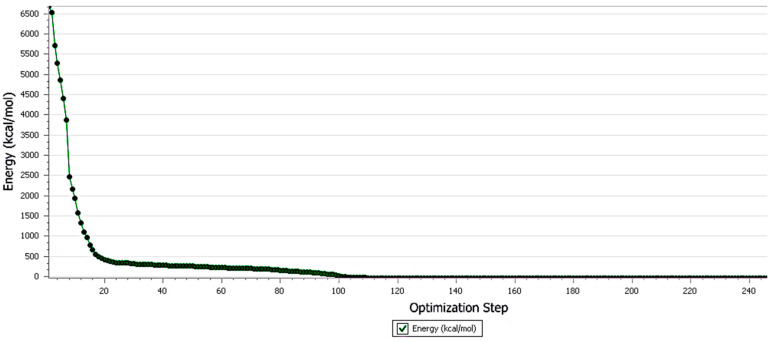
Forcite geometric optimization of acyclovir using compass forcefield.

The forcite run results showed that the geometry of the molecule and the other components was optimized and minimized to the local minimum level. The initial total energy of ACV was found to be 6683.74 kcal/mol and it was further minimized to an energy of 34.59 kcal/mol. Similarly, other molecules were also minimized to form a stable complex. The energy graph showed that the lowest confirmation molecule energy was attained at the 100th step of the process (Figure 6).

#### 3.4.1. Amorphous Cell

**Figure 7:**
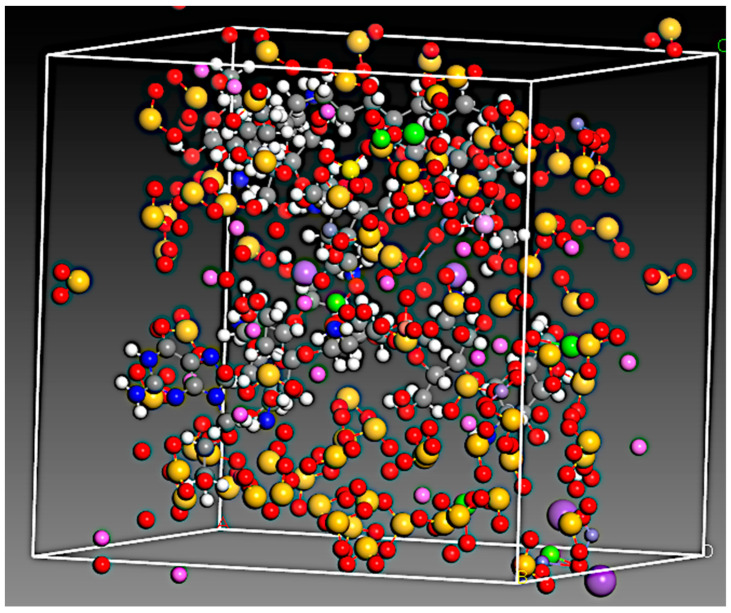
Amorphous cell with structures of the RC components, drug and polymer (where C = grey, Si = yellow, nitrogen = blue, oxygen = red, Al^3+^ = pink, hydrogen = white, iron = green).

The amorphous cell run resulted in the construction of periodic structures of the RC components, drug and polymers, which are shown in Figure 7. The 81, SiO_2_ molecules and 11, Al_2_O_3_ were placed inside the periodic boundaries with other components of RC and ACV.

**Figure 8:**
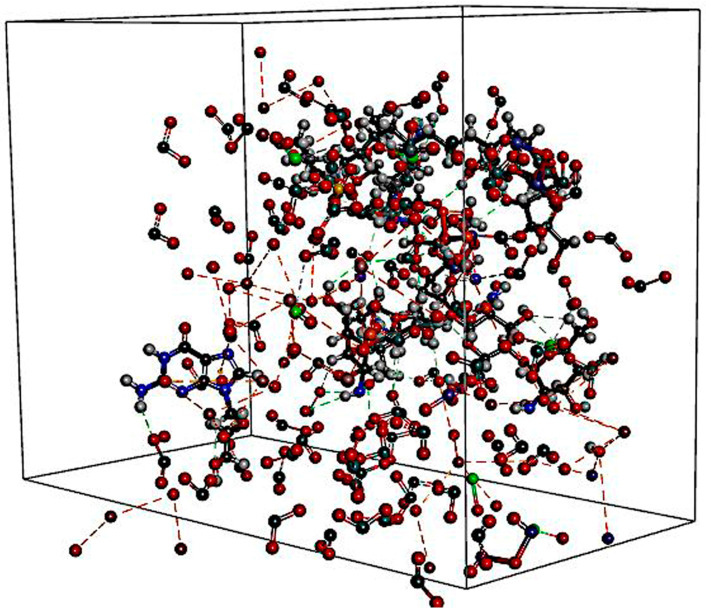
Interactions of components of the RC, polymer and drug.

**Figure 9:**
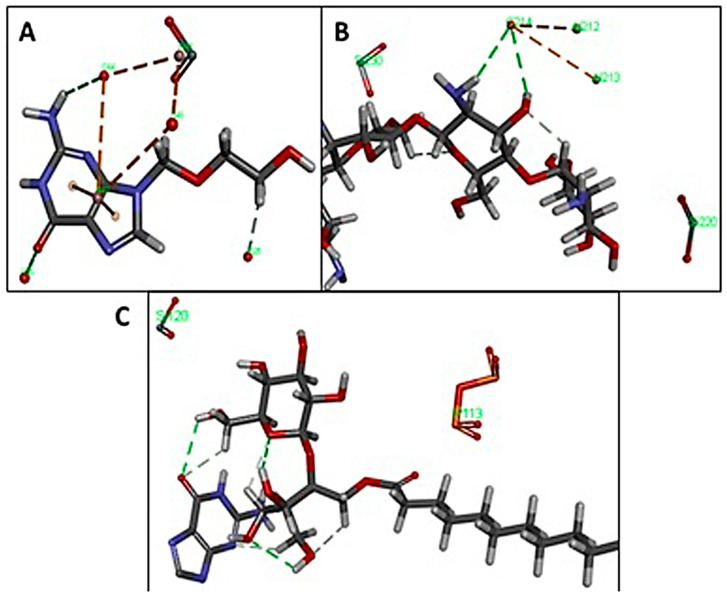
Interaction of (**A**) ACV with the Al_2_O_3_ molecule, (**B**) SS with SiO_2_ and (**C**) chitosan with ACV and SiO_2_ and P_2_O_5_. (grey: carbon; red: oxygen; blue: nitrogen)

The components formed multiple π-cationic interactions with intra-molecules. Similarly, the drug and polymers networked with RC molecules with inter- and intra-molecular non-bonded interactions. Overall, this effect makes the mixture stable (Figure 8). Particularly, ACV developed the hydrophobic interaction and hydrogen bond interactions with the Al_2_O_3_ ions (Figure 9A). SS further made the system colloidal by forming non-bonded interactions with the Si atoms of the RC (Figure 9B). Chitosan made additional stabilization through the formation of the interaction with the drug ACV and SiO_2_ atoms (Figure 9C).

#### 3.4.2. Blend Mixing

**Figure 10:**
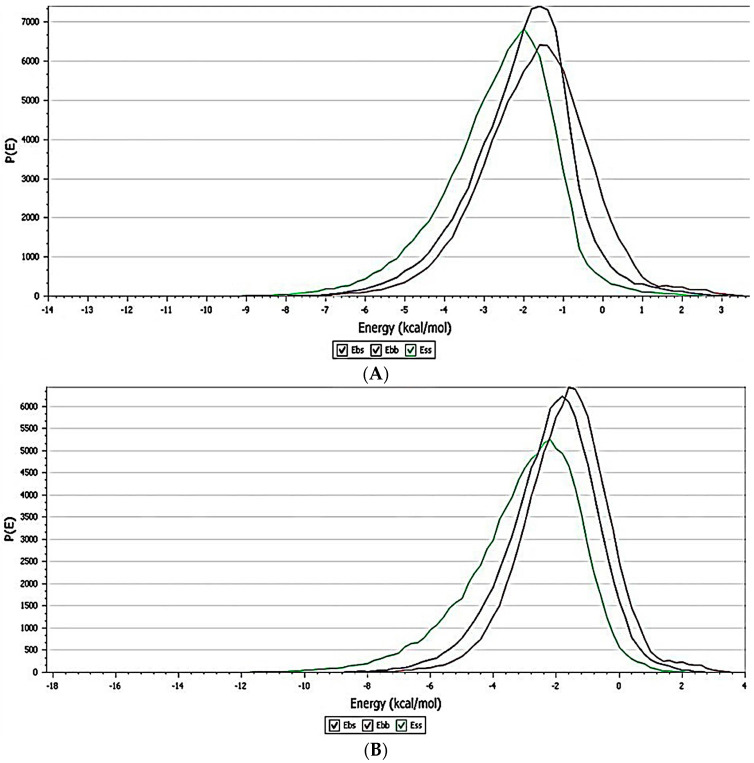
Blend binding energy distribution of (**A**) chitosan and ACV (s), (**B**) sucrose stearate and ACV(s) with RC components. Ebb = base–base (violet); Ebs = base–screen (blue); Ess = screen–screen (green).

**Figure 11:**
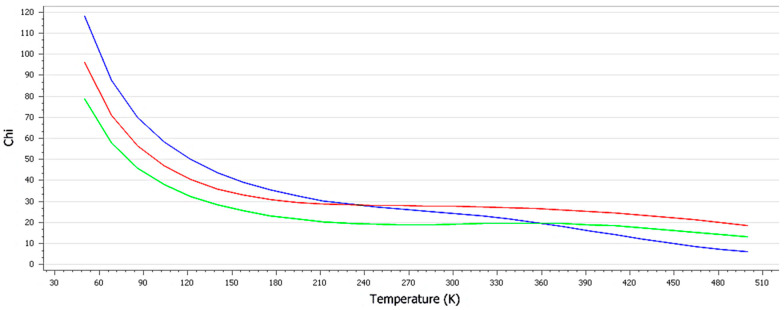
Chi value of mixing each component with red clay. The blue line represents SS, the red represents chitosan and the green represents ACV.

Blend mixing results enabled the possibilities of mixing components and energy of the lowest conformation in the mixture. The binding energy was calculated with the help of the blend algorithm. The energy distribution graph (Figure 10A,B) shows that the drug energies of the molecule and polymer are distributed equally with all components of the RC making the mixture more stable. Majorly, RC components are considered as a base (b), and the polymer and drugs are mixed as the screen(s). The blend binding energy distribution and the chi value of the system while mixing polymers and drugs are presented in Figure 10 and Figure 11, respectively.

The chi parameter or Flory–Huggins interaction parameter is used to measure the degree of interaction between blends of a polymer and solvent molecules quantitatively. Lower chi suggests a higher interaction between the polymer and solvent, thus pointing to a better stability and higher solubility of the mixture. The chi graph against the temperature confirms that the temperature did not affect the stability of the combined mixture. The three lines indicate the chi values due to the addition of SS, chitosan and ACV to RC. Based on the obtained results of the energy and interaction analysis between different modules in material studio results, it can be concluded that the mixtures of RC, the polymer and ACV are highly stable. Specifically, SiO_2_ and Al_2_O_3_ participated in the stabilization of the mixture by forming π-cationic, hydrogen and electrostatic interactions.

### 3.5. Cytotoxicity Study Using MTT

**Figure 12:**
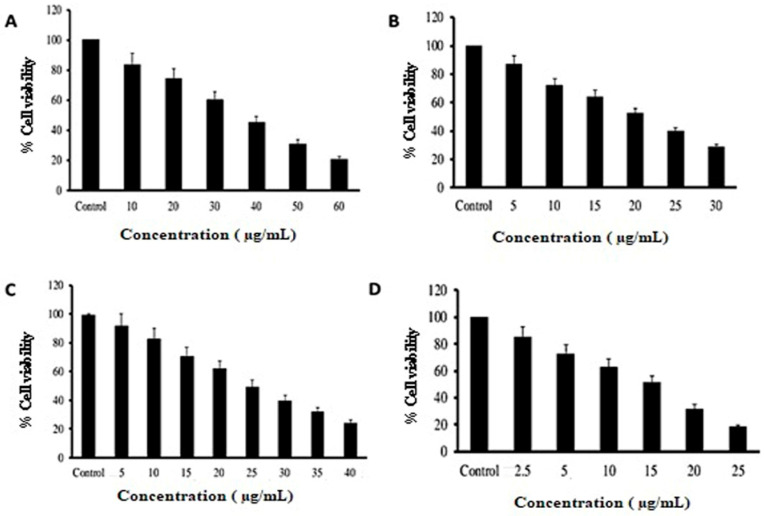
Cell viability of SK-MEL-3 cells treated for 24 h with (**A**) RC, (**B**) ACV, (**C**) RC+ACV and (**D**) DOX. The experiments were carried out in triplicates and values are expressed as mean ± SD.

The MTT assay gave valuable insight into RC acting as a cytotoxic agent either alone or along with ACV for topical drug delivery. It not only acts as a carrier molecule but also aids ACV permeation to produce its action. The results were compared with doxorubicin (DOX) as a standard drug. Figure 12 displays the cytotoxicity of the free drug and its formulation against SK-MEL-3 cells. The IC_50_ value of RC, ACV, RC+ACV and DOX was found to be 35, 20, 25 and 16 µg/mL, respectively.

### 3.6. Detection of Apoptosis with AO/EB Staining

**Figure 13:**
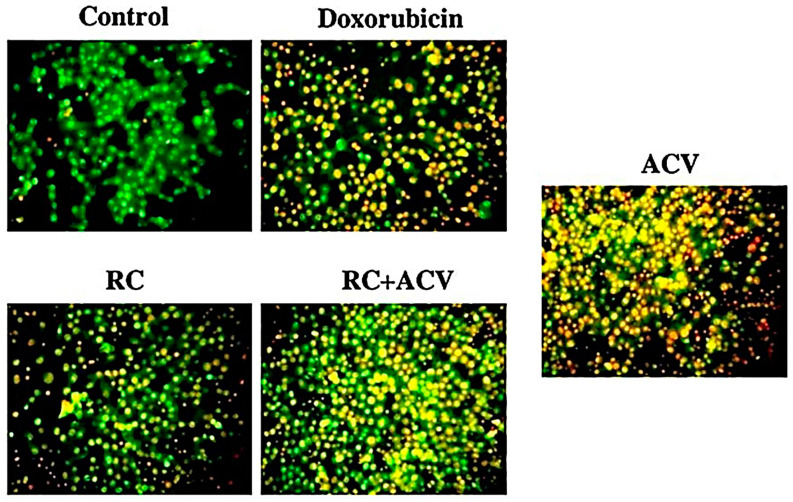
AO/EB staining of control SK-MEL-3 cells and cells treated with IC_50_ concentrations of RC, ACV, RC+ACV and DOX. Live cells appear as green, while apoptotic cells appear as yellow (early apoptotic) and green (late apoptotic).

Figure 13 displays the AO/EB staining of control SK-MEL-3 cells and cells treated with IC_50_ concentrations of RC, ACV, RC+ACV and DOX. The yellow and orange color in the cells treated with ACV, RC+ACV and DOX confirmed that the cells endured apoptosis. Morphological changes like cell shrinkage, detachment, membrane blebbing and a distorted shape were observed in treated cells.

## 4. Discussion

ACV is a well-known antiviral medication used to treat herpes simplex virus infections. However, recent studies have sparked an interest in exploring its potential for repurposing against other viral infections and even certain non-viral diseases such as cancer. Studies recently reported have demonstrated the anticancer potential of ACV towards particular cancers, making it a potential candidate for treating some types of cancer like glioblastoma. Another essential aspect of ACV repurposing is that it is an approved drug with a well-documented safety profile. Hence, it could be brought into the market faster and at a lower cost than a new drug developed from scratch. Moreover, innovative drug delivery systems like clay-based nanodrug delivery systems can address the poor physicochemical properties of ACV, limiting its applications.

Red clay-based drug delivery systems have garnered considerable attention as a promising approach for treating skin cancer. Red clay, customarily considered to be aluminosilicates rich in iron oxides, can be used as a drug delivery system with exceptional properties, including a low toxicity, better biocompatibility, biomolecule adhesion, high surface area, good absorption capacity and high ion-exchange capacity, with wide pharmaceutical applications [9]. One recently published study investigated the potential of clay as a carrier for 5-fluorouracil (5-FU) for skin cancer. The researchers prepared an Alg-CS/5-FU/Mt nanocomposite by incorporating 5-FU into red clay and evaluated its physicochemical properties and drug release behavior [17]. The findings indicated that the red clay/5-FU nanocomposites exhibited a high drug loading capacity and sustained drug release profile, thereby improving the therapeutic efficacy while minimizing the side effects of 5-FU.

The particle size analysis of clay particles showed a mean hydrodynamic diameter of 938.0 ± 97.5. The reduction in size 426.0 ± 31 nm in the final formulation, followed by the addition of SS and ACV to the suspension, confirmed the interaction between sucrose stearate and the minerals present in the red clay—further confirmed by the in silico interaction studies. Zhang et al. reported that the intercalation of kaolinite using organic solvents reduces its thickness without changing its morphology [18]. The negative charge on the surface of the nanocomplex obtained from the zeta potential value may be attributed to higher hydroxides in the RC along with negatively charged SS. RC also has various other known absorption mechanisms on its surface due to the presence of interlayers and cation exchange mechanisms [19].

The peak at 3620 cm^−1^ in FTIR indicates the presence of free OH groups in the sample. The presence of a distinct transmittance at 912 cm^−1^ indicates that nanocomplex F1 did not interact with SS or ACV. The slight stretch at 2301 cm^−1^ indicates the presence of SS on the RC surface. This leads to the further understanding that ACV binding could be in interlayers of the RC surface due to the ion exchange capacity of clay molecules. ACV is a positively charged amine group (when dissolved in dilute acid during formulation). When ACV comes into contact with clay minerals, the positively charged amine group on the drug molecule interacts with the negatively charged sites on the clay surface. This interaction leads to the exchange of ions between the clay and ACV. In the case of pure SS, a good emulsifier, the amorphous to the liquid crystalline state is attributed to the breakage of the intramolecular H-bond of sugar molecules at 222.35 °C. The liquid-to-isotropic conversion occurs at high temperatures. It helps in the solubility and permeation enhancement of ACV; hence, its poor physicochemical properties are improved by loading it into the drug delivery carrier. The nanocomplex thermogram showed a slight peak at ~280 °C and no peaks were seen at high temperatures corresponding to pure SS. This might be attributed to the role of SS in the entanglement of ACV within its crystalline liquid state and enhanced physicochemical properties of ACV [20,21].

The drug-loaded interlayer of clay is the reason for the effective encapsulation of ACV within clay nanocarriers and it is crucial for the success of clay-based drug delivery systems. The encapsulation efficiency and drug loading capacity of clay nanocarriers can be influenced by several factors, such as the type of clay used, the preparation method and the physicochemical properties of the drug. Here, the use of RC and its mineral composition is one of the key factors for a good encapsulation. Similar to our study, the anticancer drug methotrexate (MTX) was loaded within halloysite clay nanotubes (HNTs) using a solvent exchange method by Massaro et al., 2022 [22]. The researchers attributed the successful encapsulation of MTX within the HNTs to the unique structure and surface chemistry of the clay nanotubes, which allowed for an efficient drug adsorption and sustained drug release. But in our case, the release was found to be immediate and an enhanced solubility of ACV using clay and SS could be attributed to it. Moreover, in acidic conditions, the electrostatic interaction between a drug and clay is weakened, aiding in the release of the drug. At an acidic pH, the partial protonation of smectite particles may create a positive charge and reduce the surface charge of red clay and therefore the electrostatic interaction between the drug and nanoclay cleaves to aid the immediate drug release [23]. During the ion exchange process, the positively charged ions associated with the clay surface, such as sodium (Na+), calcium (Ca^2+^) or magnesium (Mg^2+^), are displaced by the ACV molecules (when exposed to an aqueous buffer, they attain a negative charge). The drug molecules bind to the clay surface through electrostatic interactions, resulting in the formation of a complex between the clay and acyclovir. The extent of ion exchange and the stability of the RC-ACV complex depend on various factors such as the pH, concentration, temperature and specific properties of RC and ACV. Hence, the exchange process is reversed, and ACV molecules are released from the clay surface under certain conditions for further action.

Clay-based drug delivery systems can provide an immediate drug release through various mechanisms, such as diffusion, swelling and erosion of the clay particles. Parallel to our study, one study investigated the immediate release of an anticancer drug, DOX, from a clay-based drug delivery system. The results showed that the DOX release from the MMT particles was controlled and reached approximately <30% within 24 h. The researchers attributed the controlled release of DOX to the layered PEG-CS/MMT sheets, which allowed the release through diffusion [24]. Furthermore, the drug release of the clay-based drug delivery system could be controlled by adjusting the concentration of the drug and the clay particles. An immediate release can be modulated to a sustained release by increasing the clay particle concentration. The reduced drug release rate indicates that the clay particles could serve as a sustained drug release platform.

When a drug is released from clay particles, it can penetrate cancer cells and exert its therapeutic effect. The drug release profile of clay-based drug delivery systems can improve the efficacy of treatment by maintaining the concentration of the drug at the tumor site. The mechanism of action of clay-based drug delivery systems in skin cancer involves several steps. First, the clay particles can penetrate the skin and reach the tumor site due to their small size and high surface area. Once they reach the site, the clay particles can adsorb or physically entrap the drug, protecting it from degradation and increasing its local concentration. In addition, the unique physicochemical properties of clay, such as its cation exchange capacity and ability to interact with proteins, can enhance the drug’s bioavailability and uptake by the cancer cells. One recently published study investigated clay nanoparticles as a potential carrier for curcumin, a natural compound with anticancer properties, for the treatment of melanoma skin cancer. The researchers prepared clay nanoparticles (Na-montmorillonite and palygorskite) loaded with curcumin that showed a good biocompatibility and were able to penetrate the skin to reach the tumor site. The researchers concluded that clay nanoparticles could be a promising platform for the delivery of anticancer drugs for the treatment of skin cancer [25].

The geometry of ACV and other components of the mixture was optimized using the COMPASS forcite protocol. Molecules at the lowest energy level (ground state) attain a stable configuration and thus form stable complexes [26]. The energy of ACV was brought down by modifying its torsional angle from 6683.74 kcal/mol to 34.59 kcal/mol in 100 iteration steps.

Clay minerals can interact with pharmaceutical organic compounds using several mechanisms. These molecular interactions are important as they can be used by formulation scientists to control the biopharmaceutical and pharmacological profile of a drug. These properties may help in increasing or decreasing the dissolution rate and immediate, delayed, extended or site-specific drug release, to reduce unwanted side effects, improve stability and for masking taste [27]. Molecular docking studies indicated that the formulated preparation was stable as the various components of RC (P_2_O_5_, SiO_2_, Al_2_O_3_), the additive, the polymer and the drug ACV formed inter- and intra-molecular non-bonding interactions. RC mineral components produced intra-molecular interactions via multiple π-cationic interactions. ACV interacted with Al_2_O_3_ ions via hydrogen bonds between NH_2_ and C=O groups in addition to van der Wall interactions (hydrophobic interactions) between the alkyl group and the neutral site on the clay. Kalonite with a composition of Al_2_Si_2_O_5_(OH)_4,_ also forms hydrogen-bonding and van der Waals interactions [28]. RC through Si atoms was seen to form non-bonding interactions with additive SS, making the system colloidal. The complex is further stabilized by polymer chitosan, which can interact with both the drug as well as P_2_O_5_ and SiO_2_ atoms of red clay.

The cytotoxic effects of the formulation on SK-MEL-3 cells were investigated with an MTT assay. The results showed a concentration-dependent reduction in the intensity of the purple color, confirming the dose-dependent decrease in cell viability, which is supported by previous reports. A previous report by Pajaniradje et al. (2014) supports our findings; they obtained a decrease in the intensity of a purple color produced as the result of the reduction in tetrazolium salts by mitochondrial enzymes [29]. ACV has an advantage over other drugs since the resistance is far less compared to other antiviral therapies for melanoma, and it produces lesser side effects. With additional red clay synergistic action, it could prove to be an effective option for the treatment of melanoma. To confirm the apoptosis, we stained the cells with AO/EB, after treatment with the IC_50_ dose of the formulation. The results showed several cells with orange, fragmented nuclei, while the untreated cells were comparatively green in color. This indicates the apoptosis induced by the formulation. Live cells appear bright green, and early to late apoptotic cells appear with yellow, orange and red nuclei [16].

## 5. Conclusions

In conclusion, our study primarily focused on the development of a drug delivery strategy specifically tailored to utilize ACV in topical treatment. Considering that ACV is the preferred antiviral medication for the treatment of a herpes virus infection, which is a major contributor to primary skin cancer leading to melanoma, the current research findings described herein hold significant implications as they can contribute to enhancing the therapeutic potential of ACV and expanding its applications in cancer treatment. Moreover, ACV also exhibited minimal side effects when compared to conventional chemotherapeutic agents like DOX.

Clay-based nanodrug delivery systems for anticancer drugs hold great promise for enhancing patient outcomes and improving the overall management of skin cancer. Overall, our findings provide a solid foundation for future research and development efforts in the field of cancer therapeutics. It can be suggested that the clay-based nanoformulation of ACV could be a better alternative to currently used conventional chemotherapeutic agents because of its ability to mitigate resistance in cancer cells.

## Figures and Tables

**Table 1:** Chemical compositions (in wt%) of red clay for in silico modeling.

Component	Percentage	Component	Percentage
CuO	0.01%	K_2_O	0.18%
ZnO	0.02%	MgO	1.57%
CaO	2.10%	Na_2_O	0.80%
P_2_O_5_	0.12%	Fe_2_O_3_	4.29%
SO_3_	0.75%	MnO_2_	0.09%
Al_2_O_3_	11.44%	SiO_2_	59.32%

**Table 2:** Particle size and zeta potential of the samples.

Sample	Zeta Potential (mV)	Particle Size (nm)
RC	−19.0 ± 7.6	938.0 ± 97.5
SS	−39.3 ± 7.9	851.0 ± 87.3
ACV	−16.9 ± 7.3	193.4 ± 20.0
Nanocomplex F1	−22.2 ± 5.1	426.0 ± 31.0

## Data Availability

The data presented in this study are available on request from the corresponding author.

## Supplementary Materials

The following supporting information can be downloaded at: https://www.mdpi.com/article/10.3390/pharmaceutics15071919/s1, Figure S1. Protocol setup for the forcite run of red clay components, polymer and drugs; Figure S2. Forcite geometric optimization set up for all the molecules; Figure S3. Amorphous cell construction protocol set up top generate the red clay realistic model; Figure S4. Blends protocol set up top generate the red clay realistic model.

## References

[1] Skin Cancer Statistics In WCRF International. https://www.wcrf.org/cancer-trends/skin-cancer-statistics/.

[2] Labani S., Asthana S., Rathore K., Sardana K. (2021). Incidence of melanoma and nonmelanoma skin cancers in Indian and the global regions. J. Cancer Res. Ther..

[3] Zhan P., Yu B., Ouyang L. (2022). Drug repurposing: An effective strategy to accelerate contemporary drug discovery. Drug Discov. Today.

[4] Pfab C., Schnobrich L., Eldnasoury S., Gessner A., El-Najjar N. (2021). Repurposing of Antimicrobial Agents for Cancer Therapy: What Do We Know?. Cancers.

[5] Taylor M., Gerriets V. (2023). Acyclovir.

[6] Shaimerdenova M., Karapina O., Mektepbayeva D., Alibek K., Akilbekova D. (2017). The effects of antiviral treatment on breast cancer cell line. Infect. Agent Cancer.

[7] Alqahtani M.S., Kazi M., Alsenaidy M.A., Ahmad M.Z. (2021). Advances in Oral Drug Delivery. Front. Pharmacol..

[8] Papich M.G., Martinez M.N. (2015). Applying Biopharmaceutical Classification System (BCS) Criteria to Predict Oral Absorption of Drugs in Dogs: Challenges and Pitfalls. AAPS J..

[9] Dong J., Cheng Z., Tan S., Zhu Q. (2021). Clay nanoparticles as pharmaceutical carriers in drug delivery systems. Expert Opin. Drug Deliv..

[10] Rakhila Y., Ezzahi A., Elmchaouri A., Mestari A. (2018). Synthesis and Characterization of a Red Clay Based New Composite Ceramic Material. Adv. Mater. Phys. Chem..

[11] (2020). Roy A French Red Clay Benefits—Skin Care Ingredients—Mirah Belle. In Magento2 Store. https://mirahbelle.com/blog/post/french-red-clay/#.

[12] Aranaz I., Alcántara A.R., Civera M.C., Arias C., Elorza B., Caballero A.H., Acosta N. (2021). Chitosan: An Overview of Its Properties and Applications. Polymers.

[13] Bernkop-Schnürch A., Dünnhaupt S. (2012). Chitosan-based drug delivery systems. Eur. J. Pharm. Biopharm..

[14] Ojeda-Hernández D.D., Canales-Aguirre A.A., Matias-Guiu J., Gomez-Pinedo U., Mateos-Díaz J.C. (2020). Potential of Chitosan and Its Derivatives for Biomedical Applications in the Central Nervous System. Front. Bioeng. Biotechnol..

[15] Mosmann T. (1983). Rapid colorimetric assay for cellular growth and survival: Application to proliferation and cytotoxicity assays. J. Immunol. Methods.

[16] Arjunan A., Pajaniradje S., Francis A.P., Subramanian S., Chandramohan S., Parthasarathi D., Sajith A.M., Padusha M.S.A., Mathur P.P., Rajagopalan R. (2021). Epigenetic modulation and apoptotic induction by a novel imidazo-benzamide derivative in human lung adenocarcinoma cells. Daru.

[17] Farshi Azhar F., Olad A. (2014). A study on sustained release formulations for oral delivery of 5-fluorouracil based on alginate-chitosan/montmorillonite nanocomposite systems. Appl. Clay Sci..

[18] Zhang Y., Long M., Huang P., Yang H., Chang S., Hu Y., Tang A., Mao L. (2017). Intercalated 2D nanoclay for emerging drug delivery in cancer therapy. Nano Res..

[19] Williams L.B., Haydel S.E. (2010). Evaluation of the medicinal use of clay minerals as antibacterial agents. Int. Geol. Rev..

[20] Sun Q., Zhang Y., Zhang Y., He H. (2017). Thermal effect on fluorine emission in coal and clay minerals. Environ. Earth Sci..

[21] Ng S.P., Khor Y.P., Lim H.K., Lai O.M., Wang Y., Wang Y., Tan C.P. (2021). Improved thermal properties and flow behavior of palm olein-based diacylglycerol: Impact of sucrose stearate incorporation. Processes.

[22] Massaro M., Poma P., Cavallaro G., García-Villén F., Lazzara G., Notarbartolo M., Muratore N., Sánchez-Espejo R., Iborra C.V., Riela S. (2022). Prodrug based on halloysite delivery systems to improve the antitumor ability of methotrexate in leukemia cell lines. Colloids Surf. B Biointerfaces.

[23] Leal D.A., Kuznetsova A., Silva G.M., Tedim J., Wypych F., Marino C.E.B. (2022). Layered materials as nanocontainers for active corrosion protection: A brief review. Appl. Clay Sci..

[24] Huang H.J., Huang S.Y., Wang T.H., Lin T.-Y., Huang N.-C., Shih O., Jeng U.-S., Chu C.-Y., Chiang W.-H. (2023). Clay nanosheets simultaneously intercalated and stabilized by PEGylated chitosan as drug delivery vehicles for cancer chemotherapy. Carbohydr. Polym..

[25] Abduljauwad S.N., Ahmed H.U.R., Moy V.T. (2021). Melanoma treatment via non-specific adhesion of cancer cells using charged nano-clays in pre-clinical studies. Sci. Rep..

[26] Senthilvel C.K., Karuppaiyan K., Pothumani A., Vedharethinam A., Jose A.W., Mohamed J.M.M., El Sherbiny M., Ebrahim H.A., El Shafey M., Dejene M. (2022). Development of Atorvastatin Calcium Biloaded Capsules for Oral Administration of Hypercholesterolemia. Evid.-Based Complement. Alternat. Med..

[27] Aguzzi C., Cerezo P., Viseras C., Caramella C. (2007). Use of clays as drug delivery systems: Possibilities and limitations. Appl. Clay Sci..

[28] Brigatti M.F., Galán E., Theng B.K.G., Bergaya F., Lagaly G. (2013). Structure and Mineralogy of Clay Minerals. Handbook of Clay Science Part A. Fundamentals.

[29] Pajaniradje S., Mohankumar K., Pamidimukkala R., Subramanian S., Rajagopalan R. (2014). Antiproliferative and apoptotic effects of *Sesbania grandiflora* leaves in human cancer cells. BioMed Res. Int..

