# Supplementary material for: Development of a Novel Red Clay-Based Drug Delivery Carrier to Improve the Therapeutic Efficacy of Acyclovir in the Treatment of Skin Cancer
Source: Pharmaceutics. 2023 Jul 10;15(7):1919. doi: 10.3390/pharmaceutics15071919 (PMC10383537; doi:10.3390/pharmaceutics15071919)
Supplement: Supplementary file 1 [file pharmaceutics-15-01919-s001.zip › pharmaceutics-2447097-supplementary.pdf]

Development of a Novel Red Clay-Based Drug Delivery Carrier to Improve the Therapeutic Efficacy of Acyclovir in the Treatment of Skin Cancer

Arul Prakash Francis [1], Aftab Ahmad [2,3], Sri Durga Devi Nagarajan [4], Harish Sundar Yogeeswarakannan [4], Krishnaraj Sekar [4], Shah Alam Khan [1,5], Dhanalekshmi Unnikrishnan Meenakshi [1,5,*], Asif Husain [6], Mohammed A. Bazuhair [7] and Nandakumar Selvasudha [8,*]

[1] Centre of Molecular Medicine and Diagnostics (COMMAND), Saveetha Dental College and Hospitals, Saveetha Institute of Medical & Technical Sciences, Saveetha University, Chennai 600077, India; fdapharma@gmail.com (A.P.F.); shahalam@nu.edu.om (S.A.K.)

[2] Health Information Technology Department, The Applied College, King Abdulaziz University,
Jeddah 21589, Saudi Arabia; abdulsalam@kau.edu.sa

[3] Pharmacovigilance and Medication Safety Unit, Center of Research Excellence for Drug Research and
Pharmaceutical Industries, King Abdulaziz University, Jeddah 21589, Saudi Arabia

[4] Department of Pharmaceutical Technology, Anna University, Chennai 600025, India; durga.elango10@gmail.com (S.D.D.N.); harishsundar2407@gmail.com (H.S.Y.); s.krishnapharma25@gmail.com (K.S.)

[5] College of Pharmacy, National University of Science and Technology, Muscat PC 130, Oman

[6] Department of Pharmaceutical Chemistry, School of Pharmaceutical Education and Research, Jamia Hamdard, Hamdard Nagar, New Delhi 110062, India; ahusain@jamiahamdard.ac.in

[7] Department of Clinical Pharmacology, Faculty of Medicine, King Abdulaziz University, Jeddah 21589, Saudi Arabia; obazohair@kau.edu.sa

[8] Department of Biotechnology, Pondicherry University, Puducherry 605014, India

* Correspondence: dhanalekshmi@nu.edu.om (D.U.M.); nkselvasudha@gmail.com (N.S.)

In silico Protocol

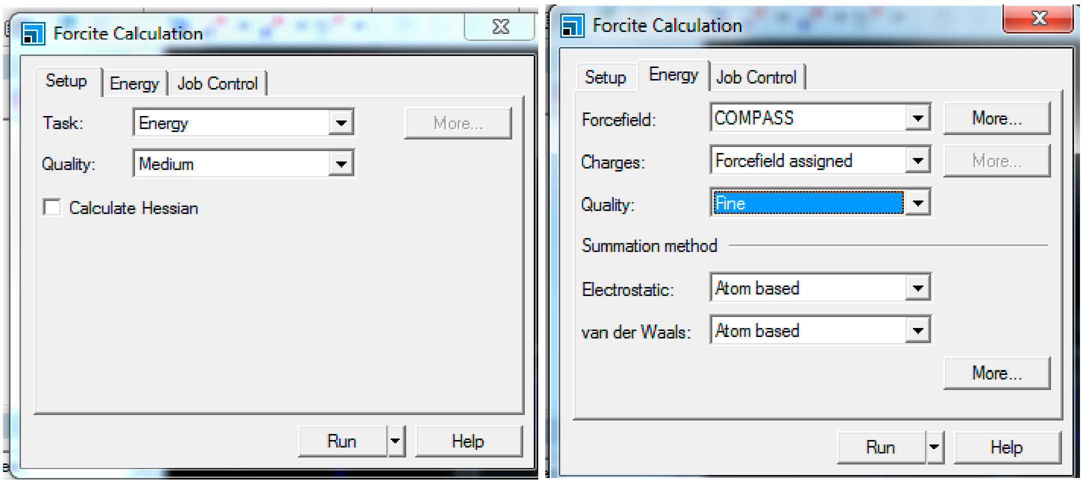

Figure S1. Protocol setup for the forcite run of red clay components, polymer and drugs

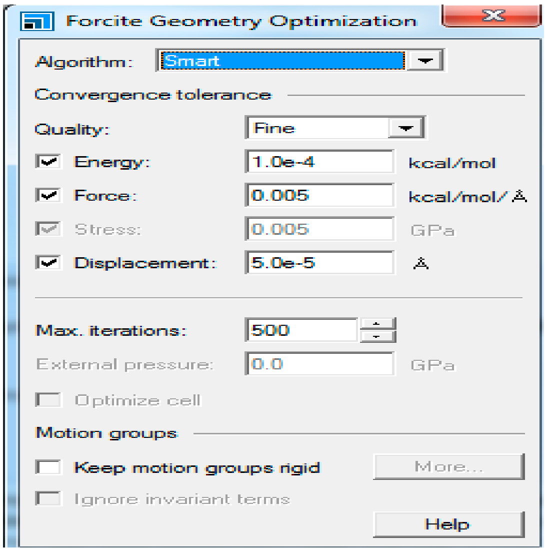

Figure S2. Forcite geometric optimization set up for all the molecules

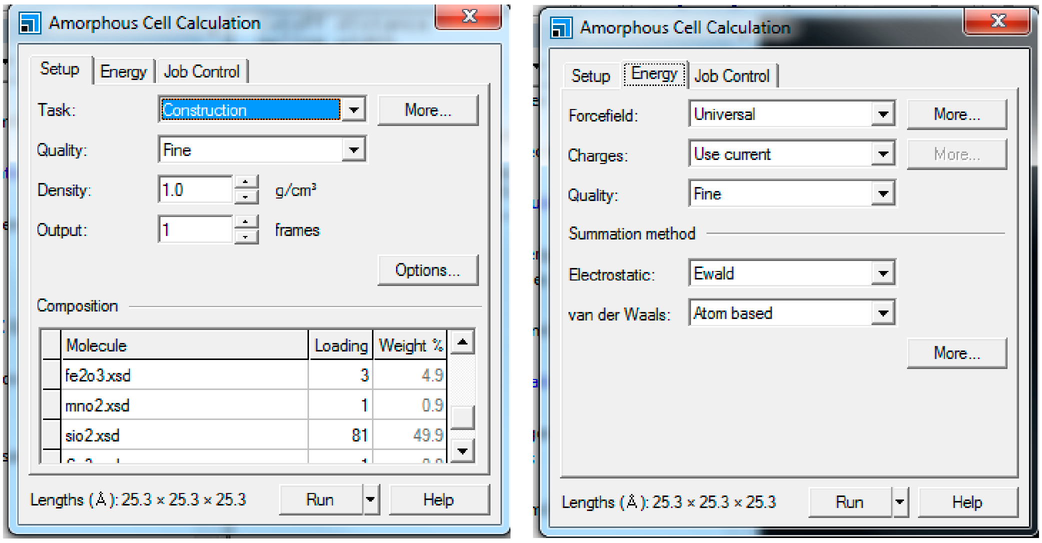

Figure S3. Amorphous cell construction protocol set up top generate the red clay realistic model.

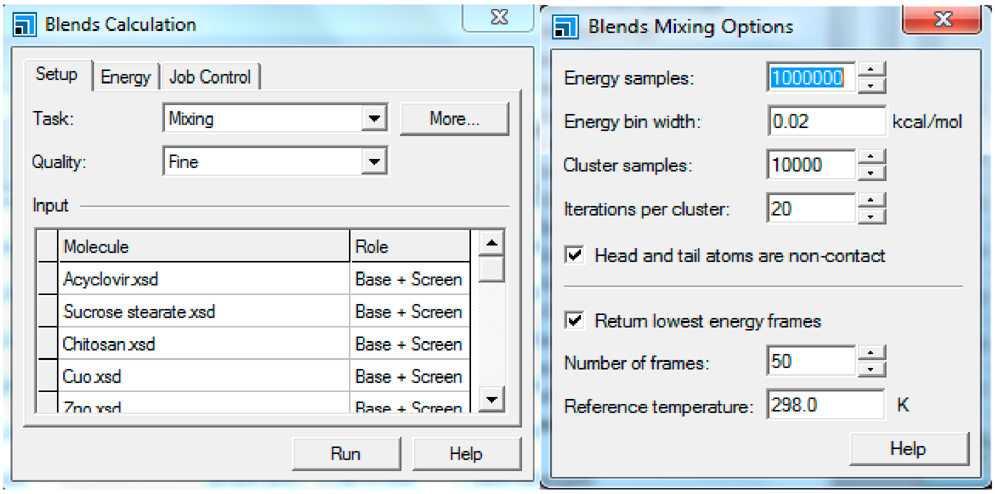

Figure S4. Blends protocol set up top generate the red clay realistic model.
